# Care-seeking and quality of care for outpatient sick children in rural Hebei, China: a cross-sectional study

**DOI:** 10.3325/cmj.2013.54.541

**Published:** 2013-12

**Authors:** Yanfeng Zhang, Qiong Wu, Michelle Helena van Velthoven, Li Chen, Josip Car, Ye Li, Wei Wang, Robert W. Scherpbier

**Affiliations:** 1 Department of Integrated Early Childhood Development, Capital Institute of Pediatrics, Beijing, China; 2 Global eHealth Unit, Department of Primary Care and Public Health, Imperial College London, London, United Kingdom; 3 Section of Health and Nutrition, Water, Environment and Sanitation, UNICEF China, Beijing, China; Zhang et al: Care-seeking and quality of care for outpatient sick children in rural China

## Abstract

**Aim:**

To assess the quality of outpatient pediatric care provided by township and village doctors, prevalence of common childhood diseases, care-seeking behavior, and coverage of key interventions in Zhao County in China.

**Methods:**

We conducted two cross-sectional surveys: 1) maternal, newborn, and child health household survey including1601 caregivers of children younger than two years; 2) health facility survey on case management of 348 sick children younger than five years by local health workers and assessment of the availability of drugs and supplies in health facility.

**Results:**

Our household survey showed that the prevalence of fever, cough, and diarrhea was 16.8%, 9.2%, and 15.6% respectively. Caregivers of children with fever, cough, and diarrhea sought care primarily in village clinics and township hospitals. Only 41.2% of children with suspected pneumonia received antibiotics, and very few children with diarrhea received oral rehydration solutions (1.2%) and zinc (4.4%). Our facility survey indicated that very few sick children were fully assessed, and only 43.8% were correctly classified by health workers when compared with the gold standard. Use of antibiotics for sick children was high and not according to guidelines.

**Conclusion:**

We showed poor quality of services for outpatient sick children in Zhao County. Since Integrated Management of Childhood Illness strategy has shown positive effects on child health in some areas of China, it is advisable to implement it in other areas as well.

Globally the number of deaths of children younger than five years decreased from 9.6 million to 7.6 million between 2000 and 2010, despite increases in the number of live births (1-3). During the past 20 years China made great achievements concerning child survival. Between 1990 and 2006, under-five mortality rate decreased from 64.6 to 20.6 per 1000 live births, and Millennium Development Goal 4 (MDG4) was achieved nine years ahead of the target set for 2015 (4-6). In 2011, under-five mortality rate was further reduced to 15.6 per 1000 live births (7). While this progress is remarkable, there remains the challenge of urban-rural mortality rate differences. Under-five mortality rate in rural areas was 2.7 times higher than in urban areas, 19.1 and 7.1 per 1000 live births, respectively (7).

Under-five mortality decrease was achieved by focusing on social development and sustained economic growth and investments in health system, including expansion of health intervention coverage (8-10). However, these were much lower in rural areas. In 2010, rural residents’ net income per capita was 5919 Yuan, which was less than one third of urban residents’ income (19 109 Yuan) (11), and the health expenditure per capita in urban areas was 3.5 times lower than in rural areas, 2316 Yuan vs 666 Yuan (7). In 2009, the number of health professionals per 1000 population was 6.03 in urban and 2.46 in rural areas, respectively (12). These factors reduce overall rural health care quality as well as the quality of pediatric care, which in rural China is often less than desirable (13-15).

To improve child survival, in the mid-1990s the World Health Organization (WHO) and United Nation’s Children Funds (UNICEF) jointly developed the Integrated Management of Childhood Illness (IMCI) strategy (16,17). The IMCI strategy has reduced the number of deaths due to diarrhea, pneumonia, malaria, measles, and malnutrition, which was estimated to 70% of all global deaths of children younger than 5 years at that time (18). IMCI has already been introduced into more than 100 countries (WHO 2005). In China it was introduced in 1998 and since 2003 has been expanded to 46 counties in 11 provinces, considerably improving health workers’ skills (19,20). Although IMCI has been in force in China for more than 10 years, training coverage remains very low for township and village doctors (21).

In 2010, the Ministry of Health of China launched a research project aiming to explore the use of appropriate medical techniques in rural areas, and IMCI was selected as a key component of the project. We carried out a household survey and a health facility survey in Zhao County, Hebei Province before IMCI implementation. The household survey aimed to assess the prevalence of common childhood diseases, care-seeking behaviors, and population coverage of key interventions, and the health facility survey aimed to assess the quality of outpatient pediatric care by township and village doctors.

## Methods

### Study design

We conducted two cross-sectional surveys in Zhao County in July and August 2011. In the first – Maternal, Newborn and Child Health (MNCH) household survey, we interviewed caregivers of children younger than two years in their households. In the second survey, an IMCI health facility survey, we directly observed the case management by township and village doctors. The two surveys will serve as the baseline data for a two-arm cluster randomized control trial with an aim to train township and village doctors IMCI.

### Survey setting

Zhao County is under the administration of Shijiazhuang City, with 16 townships and 281 villages, with a population of 571 000 and under-five population of 38 019. The county has three levels of health facilities: county hospitals (n = 4), township hospitals (n = 16), and village clinics (n = 281).

### Participants

MNCH household survey included caregivers who had a child younger than two years, while health facility survey included township and village doctors, sick children aged two months to five years, and their caregivers.

### Sample size

We based our sample size calculation on a cluster randomized control trial, which was planned to follow after the baseline surveys. For our MNCH household survey, we expected to achieve a 10% point reduction of anemia prevalence and at least a 20% point increase in knowledge and practice of appropriate feeding; and for our health facility survey, a 20% point increase in core IMCI quality of care indicators (correct classification, child not in need of antibiotics leaving the facility without antibiotics, advising the caretaker to give extra fluids and continue feeding). To obtain 80% power and 5% significance level, we calculated that for all key indicators it would be sufficient to have 800 children younger than two years in both intervention and control groups in the MNCH household survey and 192 sick children (96 from township hospitals and 96 from village clinics) in both groups in health facility survey (22).

### Sampling

We used a two-stage sampling procedure to select children and their caregivers for the household survey. First, we selected 10 clusters (villages as cluster units) in each township using proportional to population size sampling; then in each cluster, we randomly selected 13 children from the name list of all eligible children younger than two years in each village and interviewed their caregivers. The target facilities of the health facility survey were township hospitals and village clinics where the IMCI guidelines were to be implemented. We included all 16 township hospitals and randomly selected four village clinics from each township.

### Survey instruments

For the first survey, we used the MNCH household survey developed by the WHO (23). There are modules on antenatal care, delivery and neonatal care, breastfeeding and nutrition, immunization, cough and fever, diarrhea, and vitamin A. In this article, we report findings on cough, fever, and diarrhea. For the second survey, we used the WHO generic health facility survey tool (24, translated it to Chinese, and made adaptations based on the new Chinese IMCI guidelines (25). The tool has been used in China for the past ten years to evaluate the quality of care delivered to sick children attending outpatient facilities (14,20). We observed case-management using standard checklists, performed interviews with caregivers, an expert surveyor re-examined each child using IMCI technical guidelines as the “gold standard,” and we checked health facilities’ equipment and supplies.

### Survey teams and training

Three teams were formed (with one supervisor and ten interviewers per team) for the household survey and four teams (with two surveyors per team) for the health facility survey. The training for household survey lasted three days and for health facility survey one day. It included explanation of questionnaires, demonstration, and group discussions. In addition, there were communication skills explanations, role plays, and a half-day field practice in the household survey training.

### Survey methods

The household survey took place in August 2011. Data were collected using a specially developed smartphone software, described in detail in a previous study (26).

The health facility survey took place in July 2011. The teams spent a whole day in each township hospital observing 12 sick children and around two hours in each village clinic observing 3 sick children (four clinics per day). The sick children were selected according to their order of visit. One surveyor followed the case management of each child in the presence of the caregiver. Surveyors recorded the process without interfering with the health workers’ decision-making. After health worker’s diagnosis and treatment, the expert surveyor re-examined the child using IMCI guidelines. Then the first surveyor interviewed the caregivers and the expert surveyors checked equipment and supplies.

### Analysis

The household survey data were wirelessly uploaded into an Excel database. The health facility survey data were entered separately by two people using EpiData 3.1. (The EpiData Association, Odense, Denmark). The two files were compared and discrepancies resolved by referring to the original questionnaires.

Statistical analysis was performed with SAS 9.1 for Windows (SAS Institute, Cary, NC, USA) and the proportions of key indicators were reported. For the health facility survey, we followed the WHO guidelines for the main indicators analysis (21). For the drug use analysis, we manually counted the number and type of drugs and presented the proportions.

### Ethical approval and informed consent

The study was approved by the ethics committee of Capital Institute of Pediatrics. All health workers gave their written informed consent. Children’s caregivers read the information sheet and provided both oral and written informed consent on behalf of the children.

## Results

### Maternal, Newborn, and Child Health Household Survey

We interviewed 1601 caregivers of children younger than 2 years, most of them being mothers (1443, 90.1%). Among these 1601 children, 57.7% were 0-11 months and 43.3% were 12-23 months old. The boys-to-girls ratio was 1.38.

*Prevalence of common diseases.* Two-weekly prevalence of fever, cough, and diarrhea was 16.8%, 9.2%, and 15.6% respectively, whereas the prevalence of suspected pneumonia and dysentery was very low, 1.1% and 0.4% respectively (Table1).

**Table 1:** Prevalence, care-seeking behavior, and treatment of acute respiratory infections and diarrhea

	No. of children with conditions or those who received intervention (children who were eligible for the intervention)	Percentage
**Two week prevalence of:**		
Fever	269 (1601)	16.8
Cough	148 (1601)	9.2
Suspected pneumonia*	17 (1601)	1.1
Diarrhea	250 (1600)	15.6
Dysentery	7 (1600)	0.4
**Care seeking:**		
Children whose caregivers knew at least two danger signs for seeking immediate care	407 (1601)	25.4
Children with fever or cough whose caregivers sought care outside home	316 (340)	92.9
Children with suspected pneumonia whose caregivers sought care outside home	17 (17)	100.0
Children with diarrhea whose caregivers sought care outside home	211 (250)	84.4
**Treatment**		
Children with cough or fever who received drugs	275 (340)	80.9
Children with suspected pneumonia who received any antibiotics	7 (17)	41.2
Children with diarrhea who received oral rehydration therapy	160 (250)	64.0
Children with diarrhea who received oral rehydration solutions (ORS)	3 (250)	1.2
Children with diarrhea who received zinc treatment	11 (250)	4.4
Children with diarrhea who received drugs (excluding ORS)	169 (250)	67.6

*Suspected pneumonia is defined as fast breathing or difficult breathing due to chest problem.

**Figure 1:**
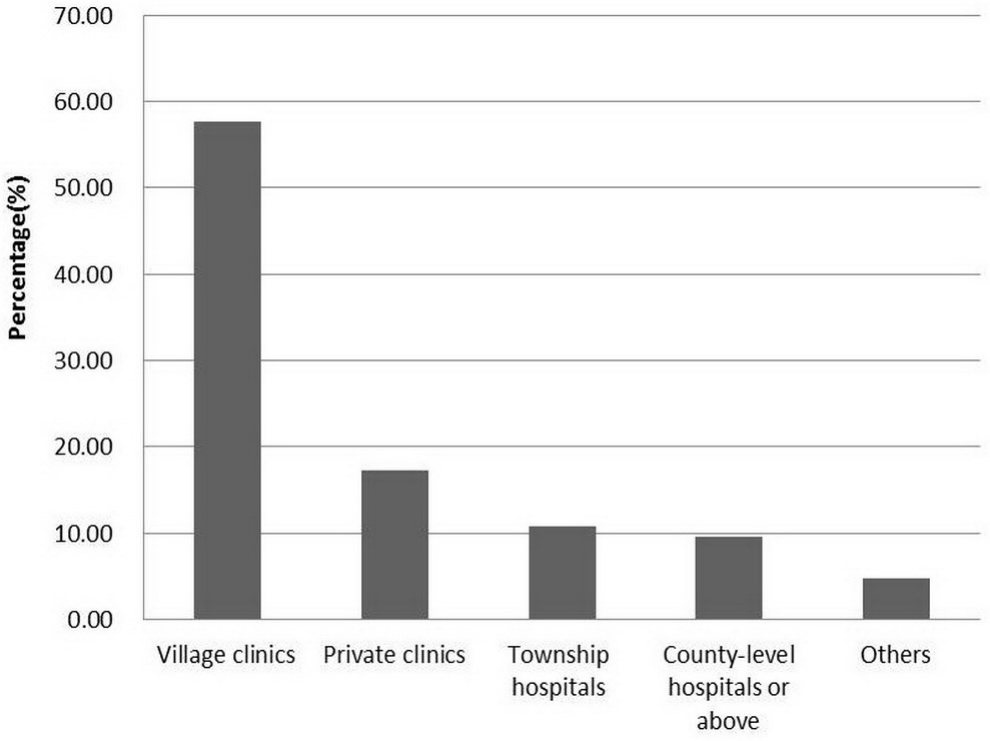
Institutions where caregivers sought care for children with fever, cough, or diarrhea.

**Figure 2:**
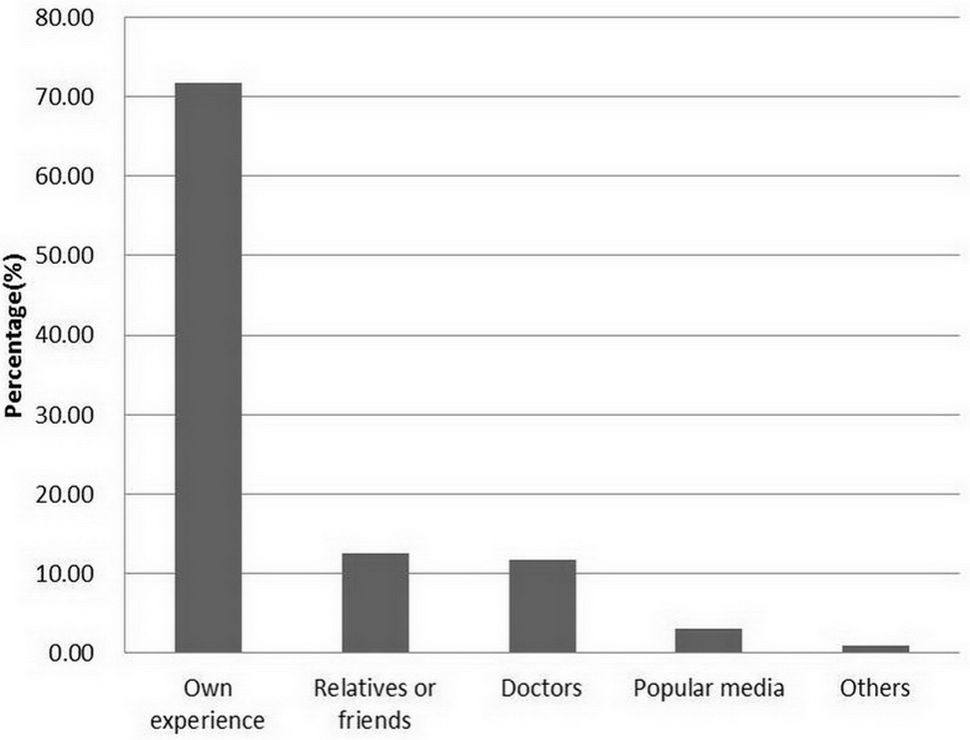
Sources of information on danger signs for caregivers.

*Care-seeking.* Only one-fourth of care-seekers knew at least two danger signs for seeking immediate care (inability to drink or be breastfed, severe vomiting, or convulsions). Most of caregivers sought care outside home when the children had fever or cough, suspected pneumonia, and diarrhea, and around two-thirds sought care in village clinics and township hospitals (Figure 1). Nearly 70% of caregivers knew the danger signs from their own experience, whereas only less than 10% heard about them from doctors (Figure 2).

*Treatment.* More than 80% of children with fever or cough received drugs, but 41.2% of children with suspected pneumonia received antibiotics. More than two-thirds of children with diarrhea received drugs, but very few of them received oral rehydration solutions (ORS) and zinc.

### Health Facility Survey

*General characteristics.* We assessed 348 sick children in 80 health facilities: 160 children in 16 township hospitals and 188 children in 64 village clinics. We were unable to recruit as many sick children as we planned to due to low caseload of some health facilities. Most of the sick children were younger than 2 years (more than 60%), and the boys-to-girls ratio was 1.9:1 (229 vs 119) (Table 2).

**Table 2:** Age and sex distribution of 348 observed sick children

	No. (%) of children
**Age in months**	
2-11	120 (34.5)
12-23	99 (28.4)
24-35	60 (17.2)
36-47	50 (14.4)
48-59	19 (5.5)
**Sex**	
Male	229 (65.8)
Female	119 (34.2)

The most frequent illness was cough or cold (44.8%), followed by diarrhea (32.8%), fever (22.4%), and anemia (6%) (Table 3). Only one severe case needed referral and 15 cases needed antibiotics (including 7 pneumonia cases, 1 severe pneumonia case, 1 dysentery case, and 6 other cases). No child suffered from dehydration (related to diarrhea) or measles (related to fever). Almost 1 in 5 cases (18.1%) had more than one disease.

**Table 3:** Symptoms or conditions of 348 sick children

	No. (%) of children
Cough or cold	156 (44.8)
Diarrhea	114 (32.8)
Fever	78 (22.4)
Anemia	21 (6.0)
Pneumonia	7 (2.0)
Low weight	2 (0.6)
Severe pneumonia	1 (0.3)
Dysentery	1 (0.3)
Other*	33 (9.5)
Single classification	285 (81.9)
Multi-classifications	63 (18.1)

*Tonsillitis, apthous stomatitis, and skin pustular eruption.

*Assessment and classification.* The overall assessment index was 18 out of 100 (indicating very poor assessment) (Table 4). Only about 1 in 10 children (10.9%) were checked for the presence of cough, diarrhea, and fever. Even fewer children (4.9%) were weighed, and only one child was checked against a growth chart. Very few children (4.6%) were checked for vaccination status, and only 2 caregivers were asked about the feeding practices. None of the children were checked for three danger signs. Moreover, classification was poor, as less than a half of the children (43.8%) were correctly classified.

**Table 4:** Proportion of children and caregivers for whom specific case-management tasks were performed by health providers

Tasks according to IMCI guidelines	No. (%) of children or caregivers eligible for task based on expert's recheck*	No. (%) of children or caregivers in whom the task was performed based on assessor's observation^†^
**Assessment**		
Index of integrated assessment (mean) (range 0-100)	-	18
Weighing	348 (100.0)	17 (4.9)
Taking body temperature	348 (100.0)	210 (60.3)
Checking for three danger signs	348 (100.0)	0 (0.0)
Checking for the presence of cough, diarrhea, and fever	348 (100.0)	38 (10.9)
Checking against the growth chart	348 (100.0)	1 (0.3)
Checking the vaccination status	348 (100.0)	16 (4.6)
Asking the caregiver about feeding practices (for children younger than 2 y)	219 (62.9)	2 (0.9)
Checking for other problems	348 (100.0)	104 (29.9)
**Classification**		
Correct classification	348 (100.0)	152 (43.7)
**Treatment**		
Pneumonia treated by antibiotics	7 (2.0)	3/7^‡^
Correct prescription of oral antibiotic	8 (2.3)	4/8^‡^
The child who needed oral antibiotics received the first dose of treatment at the facility	8 (2.3)	6/8^‡^
The child who did not need antibiotic was not given them	332 (95.4)	198 (59.6)
Oral rehydration solutions and zinc for diarrhea	114 (32.8)	0 (0.0)
Referral given to the child who needs it	1 (0.2)	0 (0.0)
**Advice and counseling given to caregivers**		
To give extra fluids and continue feeding	347**^§^** (99.7)	2 (0.6)
On how to give prescribed oral antibiotics	96 (27.6)	92 (95.8)
On when to return to the doctor’s immediately	347**^§^** (99.7)	0 (0.0)

*Among all surveyed children, these children or their caregivers should be given specific case-management tasks by health providers based on the expert's recheck.

†Among those children or their caregivers who should be given the specific case-management tasks, these were actually given by health providers.

‡No percentage given as the numbers are very small.

§One child needing referral was not eligible for advice and counseling according to IMCI guidelines.

*Treatment.* Many children were not given the necessary medications, were treated incorrectly, or were given unnecessary treatment. None of the 114 children with diarrhea received ORS and zinc. Less than half of pneumonia cases were treated correctly (3 out of 7). Half of sick children needing oral antibiotic were prescribed the drug correctly (4 out of 8). Out of children who did not need antibiotics, 59.6% were not given antibiotics.

*Advice and counseling* Nearly all caregivers of children (95.8%) who were prescribed oral antibiotics were advised on their proper administration. However, only two caregivers were advised to give extra fluids and continue feeding, and none was advised on when to seek care immediately.

*Drug use*. The mean number of drugs given to each sick child was 1.7 (609/348, range 0-5) and more than one-fifth of children (22.4%, data not shown in the table) were given three different types of drugs. A total of 150 children (43.1%) were given antibiotics, but only 15 (4.3%) required antibiotics according to the gold standard assessment (Table 5). The most frequently used antibiotics were first generation cephalosporins (15.8%), followed by third generation cephalosporins (8.9%), aminoglycosides (7.2%), macrolides (6.3%), amoxicillin (4.6%), and others (2.0%). Nearly 1 in 5 children (19.5%) were given antivirals, and ribavirin was the most commonly used antiviral (14.7%). Forty-nine children (14.1%) were given antidiarrheals; smectite was the most commonly used antidiarrheal (12.6%). Sixty-five cases (18.7%) were given antipyretics; single ibuprofen or paracetamol products were rarely used (2.9%), compound amidopyrin injection was given to 4.3% of children intramuscularly, and more than 10% of children were given compound preparation containing paracetamol. Many children (54.0%) were given traditional Chinese drugs, which included pure traditional Chinese drugs (37.9%) and compound preparations combining traditional Chinese drugs and chemical drugs (21.6%). Only in one severe pneumonia case antibiotics were given parenterally and in 53 cases (15.2%) they were given intramuscularly or intravenously (data not shown in the table).

**Table 5:** Use of different types of drugs

Types of drugs	No. (%) of children
Antibiotics	150 (43.1)*
First generation of cephalosporin	55 (15.8)
Third generation of cephalosporin	31 (8.9)
Aminoglycosides	25 (7.2)
Macrolides	22 (6.3)
Amoxicillin	16 (4.6)
Other	7 (2.0)
**Antivirals**	**68 (19.5)^†^**
Ribavirin	50 (14.7)
Compound preparation	7 (2.0)
Other	3 (0.9)
**Antidiarrheals**	**49 (14.1)**
Smectite	44 (12.6)
Other	5 (1.4)
**Antipyretics**	**65 (18.7)**
Ibuprofen/paracetamol	10 (2.9)
Antiondin (intramuscular)	15 (4.3)
Compound preparation	37 (10.6)
Other	3(0.9)
**Traditional Chinese drug**	**188 (54.0)^‡^**
Pure traditional Chinese drug	132 (37.9)
Compound preparation	75 (21.6)

*Six sick children were given two antibiotics.

†Two sick children were given both ribavirin and compound preparation.

§Nineteen sick children were given both pure traditional Chinese drug and compound preparation.

*Availability of drugs and supplies*. All health facilities were supplied with functioning weighing scales. The availability of drug supplies varied considerably. Drugs that were in good supply (more than 80%) were ceftriaxone, iodophor, acetaminophen, vitamin A and D, vitamin B complex, erythromycin ophthalmic ointment, albendazole, azithromycin, and Trimethoprim-Sulfamethoxazole combination drugs. Drugs in poor supply (less than 50%) were amoxicillin, ofloxacin ear drops, zinc, diazepam, salbutamol aerosol, and ORS. The index of availability of the recommended drugs was 61 out of 100 (Table 6).

**Table 6:** The availability of specific supplies and recommended drugs in 16 township hospitals and 61* village clinics (n = 77)

	No. (%) of township hospitals and village clinics having specific supplies and recommended drugs
Functioning child weighing scale	16 (20.0)
Functioning adult weighing scale	62 (80.5)
Ceftriazone	77 (100.0)
Iodophor	74 (96.1)
Acetaminophen	72 (93.5)
Vitamin A	12 (15.6)
Vitamin A/D	71 (92.2)
Vitamin B complex	71 (92.2)
Erythromycin ophthalmic ointment	70 (90.9)
Albendazole	68 (88.3)
Azithromycin	67 (87.0)
Trimethoprim+Sulfamethoxazole	61 (79.2)
Iron	39 (50.7)
Amoxicillin	37 (48.1)
Ofloxacin ear drops	35 (45.5)
Zinc	29 (37.7)
Diazepam	11 (14.3)
Salbutamol aerosol	7 (9.1)
Oral rehydration solutions	3 (3.9)
Mean availability index of recommended drugs (range 0-100)	61.2 (35.3-94.1)

*Information on three village clinics was missing.

## Discussion

The maternal, newborn, and child health household survey showed high prevalence of fever, cough, and diarrhea and low prevalence of suspected pneumonia and dysentery. Caregivers primarily sought care for children with fever, cough, and diarrhea in village clinics and township hospitals. Only a small proportion of caregivers knew the danger signs for seeking immediate care. The health facility survey showed the need for improvement of case management skills of rural doctors as the assessments, classification, and treatment of sick children was often incorrect.

The most common childhood diseases in China are acute respiratory infection and diarrhea. According to the national data for 2008, the two-weekly prevalence of all diseases for children under five was 17.4%, with acute respiratory infection and diarrhea accounting for 65.2% and 13.5% of cases, respectively (27). Our data from household survey also indicated high prevalence of fever (16.8%), cough (9.2%), and diarrhea (15.6%). In addition, pneumonia is still the leading cause of under-five mortality, accounting for 16.5% of the total deaths, while diarrhea contributes to 3.0% of total under-five mortality (6). Our data showed that caretakers of most children with fever, cough, or diarrhea sought care outside home in village clinics and township hospitals. A study in 10 western provinces found similar results: more than 80% of children with common cold and diarrhea were brought to township hospitals or village clinics (nearly 50% were brought to village clinics) (28). However, the quality of outpatient care for sick children in these health facilities was lower than recommended in IMCI guidelines.

The problem of poor case management skills of rural doctors is not limited only to our study area. A study in Yunnan Province using the same WHO health facility survey instrument also found inadequate history taking and physical examination, inability to detect potentially serious complications, over prescription of injection and antibiotics, and under prescription of ORS and poor quality of counseling by village doctors (15). An exploratory study in China also showed that cough, cold, and diarrhea were often incorrectly treated with antibiotics (29). The incomplete assessment of a sick child may lead to delays in diagnosis and treatment, incorrect classification may lead to incorrect treatment, and inappropriate use of drugs may harm children (30-32). Also, this all burdens families and health system, and increases health care expenditure (33,34).

At the end of 2011, there were 37 295 township hospitals with 981 000 health professional workers, and 662 894 village clinics with 1 126 000 health professional workers all over China. The total caseload per year per township hospital and per village clinic was 23 000 person times and 2700 person times, respectively (7). Township hospitals and village clinics are the main health care service providers for rural population, but the provided services are generally of poor quality. Health workers in these facilities are often not well educated. More than 60% of township health professional workers did not attend university and had only paramedical school, junior high school, or lower education level, and 94% of village doctors had only paramedical school or lower education level (12). Their diagnosis and treatment of diseases are usually based on out-of-date guidelines and experience rather than updated evidence-based guidelines. Other studies showed that many physicians believed that antibiotics or antivirals could facilitate a speedy recovery (29) and anti-diarrheal drugs should be given to children with diarrhea (35). In addition, traditional Chinese medicines are very commonly used in children. Similar to our results, studies reported that 64.2% of sick children were given traditional medicines and many doctors used both chemical medicines and traditional medicines that have the same effect (36). This puts additional economic burdens to parents and potentially harms the children, since more and more adverse effects of traditional medicines are being reported (36,37).

Our study had some limitations. First, it was carried out in one county over a short period of time, which makes the results representative only for settings with similar characteristics. Second, the presence of our surveyors may have affected the quality of care offered by township and village doctors. However, we do not believe that this considerably influenced our findings since it is unlikely that doctors would have provided better care in our absence.

Our study showed poor quality of the services provided to outpatient sick children in Zhao County, Hebei Province in China. Similar findings were also observed in other areas, indicating a need for improvement of the care provided by rural doctors. IMCI technical guidelines are soundly evidence-based (38-40) and appropriate for settings with limited resources. Since they have shown positive effects on child health in some areas of China, it is advisable to implement them in other areas as well.
